# CVR-BBI: an open-source VR platform for multi-user collaborative brain to brain interfaces

**DOI:** 10.1093/bioinformatics/btae676

**Published:** 2024-11-22

**Authors:** Di Liu, Yina Wei

**Affiliations:** School of Biological Science and Medical Engineering, Southeast University, Nanjing 210096, China; Research Center for Frontier Fundamental Studies, Zhejiang Lab, Hangzhou 311100, China; Department of Biomedical Engineering, Zhejiang University, Hangzhou 310027, China

## Abstract

**Summary:**

As brain imaging and neurofeedback technologies advance, the brain-to-brain interface (BBI) has emerged as an innovative field, enabling in-depth exploration of cross-brain information exchange and enhancing our understanding of collaborative intelligence. However, no open-source virtual reality (VR) platform currently supports the rapid and efficient configuration of multi-user, collaborative BBIs. To address this gap, we introduce the Collaborative Virtual Reality Brain-to-Brain Interface (CVR-BBI), an open-source platform consisting of a client and server. The CVR-BBI client enables users to participate in collaborative experiments, collect electroencephalogram (EEG) data, and manage interactive multisensory stimuli within the VR environment. Meanwhile, the CVR-BBI server manages multi-user collaboration paradigms, and performs real-time analysis of the EEG data. We evaluated the CVR-BBI platform using the SSVEP paradigm and observed that collaborative decoding outperformed individual decoding, validating the platform’s effectiveness in collaborative settings. The CVR-BBI offers a pioneering platform that facilitates the development of innovative BBI applications within collaborative VR environments, thereby enhancing the understanding of brain collaboration and cognition.

**Availability and implementation:**

CVR-BBI is released as an open-source platform, with its source code being available at https://github.com/DILIU1/CVR-BBI.

## 1 Introduction

The advancements in brain imaging and neurofeedback technologies have driven the demand for innovative approaches to regulate and analyze brain activities. Brain-to-brain interface (BBI) technology, which combines brain-computer interface (BCI) and computer-brain interface (CBI), emerges as a novel approach for investigating information exchange between brains, including even cross-species communication (Gao *et al.* 2021). Recent studies have shown that collaborative activities within groups can synchronize brain activity patterns among participants (Jia *et al.* 2024), highlighting the importance of collaboration in exploring cooperative brain functions and optimizing paradigms. VR environments significantly enhance users’ training capabilities and attention compared to non-VR exercises (Ko *et al.* 2020), emphasizing the significance of virtual reality environments in exploring brain potential and enhancing intelligence (Zhang *et al.* 2023). In addition, BBI has been utilized in the medical rehabilitation of patients with depression (Li *et al.* 2021).

In this study, we introduce CVR-BBI, an open-source VR platform consisting of both a client and a server, designed to facilitate the rapid and effective development of innovative multi-user collaborative BCIs by scientists. The client is designed to create and manage interactive multisensory stimuli within virtual environments. It supports the collection of EEG data from users interacting within the virtual space. The server processes multi-user collaboration messages, offering real-time analysis and closed-loop feedback of EEG data. This study provides an intuitive graphical user interface for multi-user collaborative BBI, which will further enhance our understanding of brain functions and brain intelligence.

## 2 Methods and implementations

**Figure 1.**
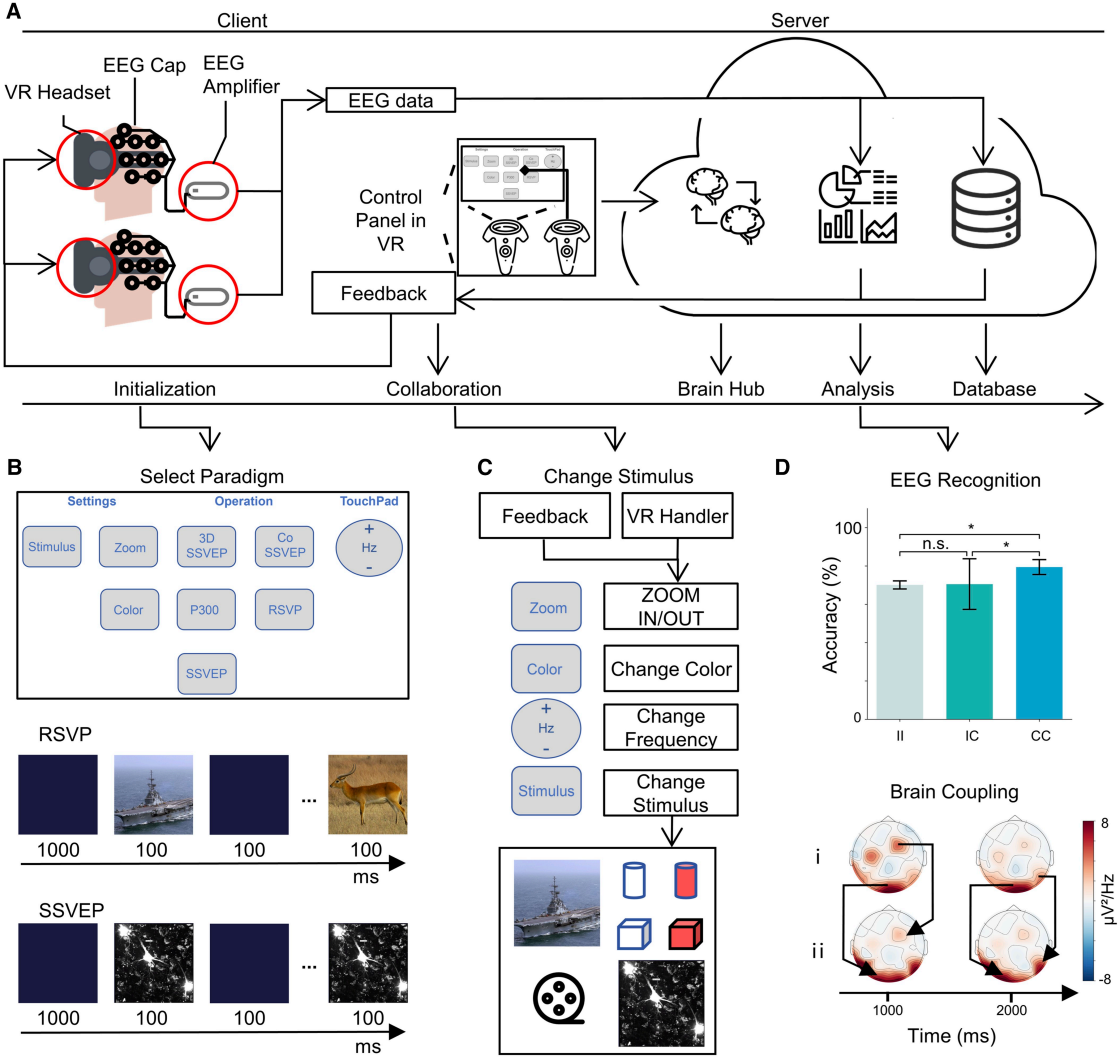
The framework of the CVR-BBI Platform. (A) The overview of the CVR-BBI platform. The client transmits EEG data to the server and, in turn, receives analytical feedback. Meanwhile, the server conducts real-time analysis of the received EEG data, and concurrently archives the associated stimuli, EEG signals, and the outcomes of the analysis. (B) Experimental paradigms. Through the functional selection panel in VR, choose the paradigm of interest. The RSVP and SSVEP paradigms are demonstrated. (C) The stimulus configuration. Four types of stimuli are presented, differentiated by factors such as form, color, size, and flickering frequency. (D) EEG offline analysis. Top: comparison of SSVEP recognition accuracies among groups: individual paradigms with individual decoding (II), collaborative paradigms with individual decoding (CI), and collaborative paradigms with collaborative decoding (CC). Due to the limited sample data, we used Cohen’s *d* effect size to measure the differences between groups. * represents small difference (|*d*|>0.2). n.s. represent no difference. Bottom: brain topography maps display brain coupling when two individuals simultaneously engage in the SSVEP task.

The CVR-BBI, developed from the established Collaborative Augmented Reconstruction (CAR) platform, enables immersive interaction and collaborative processes across various client devices. It consists of two modules: a client and a server (Fig. 1A). After installing the client, it is crucial to ensure that the VR and EEG device drivers, compatible with the HTC Vive Pro and ANT Neuro eego™ amplifier (Supplementary Fig. S1), are properly installed. For experiments requiring remote multi-user collaboration, the server must be deployed on a remote server equipped with a public IP, such as those provided by Amazon Cloud or Tencent Cloud. For rapid deployment and reproducibility, we have prepared a Docker image that includes the CVR-BBI server along with all necessary tools and dependencies. The detailed instructions for launching a demo to collect EEG data are available in the GitHub repository (Supplementary Figs S2 and S3).

### 2.1 CVR-BBI client

The VR client, developed in C++, utilizes its robust performance, cross-platform capabilities, and rich library resources to facilitate the rendering of VR stimulation and the simultaneous acquisition of EEG signals. During experiments on the platform, multisensory stimuli are generated and displayed in real time within the VR environment, while EEG signals are simultaneously collected and sent to the server for analysis. The CVR-BBI client supports a VR refresh rate of up to 90 Hz and an EEG sampling frequency of up to 1 kHz. Subsequently, the client receives the results of the data analysis from the server, using it to dynamically adjust the visual properties of the stimuli, such as color, size, and type of stimuli within the VR settings, to achieve closed-loop feedback on brain activity. This experimental protocol ensures real-time interaction between the virtual stimuli and the user’s brain responses, enhancing the effectiveness of the experiment. The client is designed to display various sensory stimuli, including visual stimuli such as natural images, biomedical images, and geometric structures (Supplementary Fig. S4), as well as auditory stimuli. It supports stimulus rendering in paradigms such as visual reconstruction, task collaboration, and semantic understanding. The client provides collaborative paradigms for Steady-State Visually Evoked Potential (SSVEP) and Rapid Serial Visual Presentation (RSVP) (Fig. 1B). Users have the capability to create new C++ scripts based on the client template, enabling the design of customized multi-user collaborative paradigms within the VR environment. The client’s modular programming architecture allows the developers to efficiently and promptly transmit data to the server and obtain real-time analysis outcomes through its data transfer interfaces. For deploying multi-user collaborative paradigms, it is essential to design real-time brain feedback and EEG data analysis, following the paradigm design procedure (Supplementary Fig. S5).

### 2.2 CVR-BBI server

The CVR-BBI server is structured into two primary sections: the collaboration module and the data analysis module. The collaboration module supports real-time participation in EEG experiments by multiple users and manages data exchange between clients. It handles the initiation and termination of collaboration paradigms, synchronization of stimulation modes, and transmission of complex collaboration messages. In the collaborative VR environment, the average response time for collaborative messages is <0.27 ms (Zhang *et al.* 2023). The modular code structure and well-encapsulated program design (Supplementary Fig. S5), allow developers to integrate custom code tailored to specific collaboration logic on both the client and server. The data analysis module, developed using the Django framework, is designed for the robust online processing of EEG signals. It supports various data input formats, including file and binary types. When EEG data is transmitted to the data analysis module, the module invokes customized algorithms based on different routing addresses to conduct an in-depth analysis of the EEG data. This analysis includes EEG preprocessing, EEG classification (Gu *et al.* 2024), brain coupling analysis (Wang *et al.* 2019), and neural decoding (Grootswagers *et al.* 2022). Once data analysis is completed, the server transmits the real-time results back to the client, enabling closed-loop feedback and targeted brain stimulation of specific regions (Fig. 1C).

### 2.3 Environmental configurations

The CVR-BBI platform, built on the CAR platform (Version 1.3.2), is designed for installation on Windows 10. To use the HTC Vive Pro VR device, the SteamVR software (Version 2.7.4) is required. The platform is also compatible with EEG device drivers for the ANT Neuro eego™ amplifier. All experiments were performed on a computer with a high-performance configuration, including an Intel Core i9-10900K CPU @ 3.70 GHz, 64 GB of RAM, and an NVIDIA GeForce GTX 2080 GPU.

### 2.4 Data analysis

We evaluated the CVR-BBI platform using the SSVEP paradigm, collecting data from five healthy individuals engaged in both individual and collaborative experiments. Each experiment included three stimulus targets at frequencies of 8, 10, and 13 Hz, with each target comprising 10 trials conducted twice. The data were downsampled to 512 Hz and filtered at 4–45 Hz frequency range, and then segmented into 1-s epochs. We estimated the power spectral density (PSD) of each channel using the Welch method. For individual decoding, power was averaged across the O1, O2, and Oz channels of each participant, while for collaborative decoding, power was averaged across the same channels of all participants. The frequency with the highest power in each segment was identified to compute the recognition accuracy of the SSVEP paradigm.

To assess the brain synchrony in collaborative experiments, we performed a brain coupling analysis. We first extracted PSD at the stimulus target frequency for each channel at 1-s EEG trial of collaborative participants. The Pearson correlation coefficients were computed between the PSD of all channels for each pair of participants in each trial, then standardized using Fisher’s Z transformation. Next, we calculated the mean Fisher Z value of correlation coefficients across all 10 trials and derived an overall *P*-value from this mean Z value based on the normal distribution. This *P*-value indicates the statistical significance of the observed brain coupling, determining whether the synchrony is significantly greater than chance.

## 3 Results

To assess the platform’s efficiency for real-time analysis, we performed an online processing test that involved preprocessing and computing the PSD of SSVEP signals collected from 32 channels, with each channel having 1000 data points. The platform’s online processing time was <30 ms, demonstrating its efficiency in handling real-time data analysis tasks.

In the offline analysis, we compared the SSVEP recognition accuracy among three groups: individual paradigms with individual decoding (II, *n* = 6), collaborative paradigms with individual decoding (CI, *n* = 16), and collaborative paradigms with collaborative decoding (CC, *n* = 8). The findings reveal that the recognition accuracy in collaborative paradigms, when using collaborative decoding, outperforms that of individual decoding (Fig.1D, top). This result validates the platform’s effectiveness and reliability in collaborative settings.

We also conducted a brain coupling analysis to assess the degree of synchrony among participants during the collaborative tasks. Specifically, we found a correlation coefficient of 0.77 (±0.15) between participants at the 8 Hz stimulus target frequency during the 10-s SSVEP tasks (Fig. 1D, bottom, *P* < 0.001). This high correlation indicates a significant level of synchrony between brain regions during the collaborative task. These findings further validate the platform’s effectiveness during collaborative interactions.

## 4 Conclusions

CVR-BBI offers an open-source VR platform for collaborative BBI experiments, designed for EEG data collection and closed-loop neural feedback in collaborative paradigms. With its extensive paradigm support and modular program design, CVR-BBI enables users to efficiently design multi-user collaborative paradigms within VR environments for brain science.

However, the platform does face certain potential limitations, such as inherent latency in real-time EEG data processing and user experience issues such as VR-induced discomfort. To address these challenges, our future work will focus on optimizing algorithms to minimize latency and refining the VR environment to improve user comfort. In addition, the platform can be integrated with advanced neuroimaging, including electrocorticography (ECoG) and stereoelectroencephalography (SEEG), along with neuromodulation techniques such as transcranial magnetic stimulation (TMS) and deep brain stimulation (DBS). This integration could potentially position CVR-BBI as a versatile tool in both cutting-edge neuroscience research and clinical applications.

## Supplementary Material

btae676_Supplementary_Data

## Data Availability

The data underlying this article are available in CVR-BBI repository at https://github.com/DILIU1/CVR-BBI.
