# Supplementary material for: CVR-BBI: an open-source VR platform for multi-user collaborative brain to brain interfaces
Source: Bioinformatics. 2024 Nov 22;40(12):btae676. doi: 10.1093/bioinformatics/btae676 (PMC11629691; doi:10.1093/bioinformatics/btae676)
Supplement: btae676_Supplementary_Data [file btae676_supplementary_data.docx]

# CVR-BBI Supplementary Information


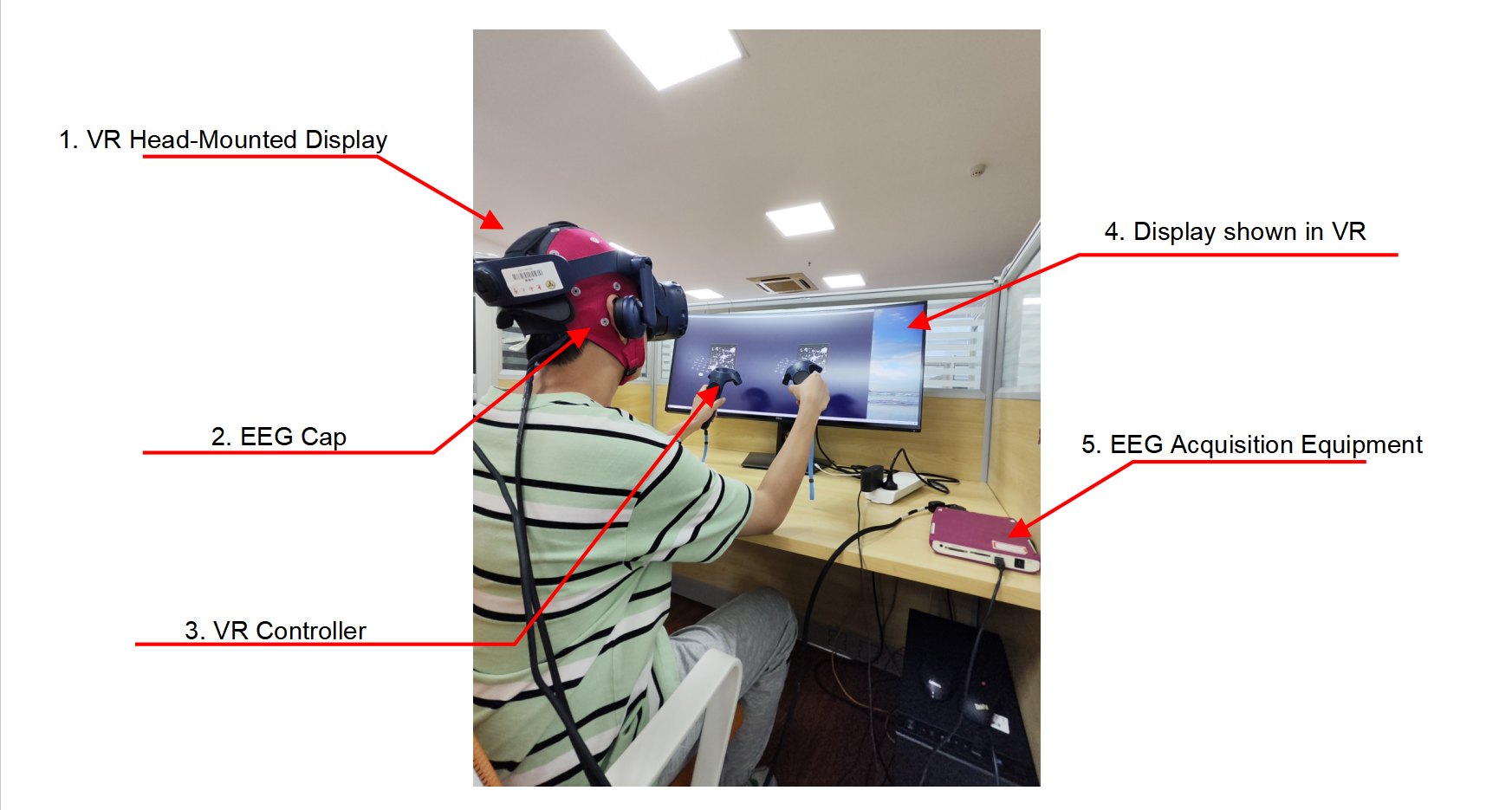


**Fig. S1. The** **Hardware Configuration for CVR-BBI.** **1) VR Head-Mounted Display (VR HMD).** It offers participants a fully immersive 360-degree visual experience, essential for simulating real-world or multisensory stimuli within the virtual environment. **2) Electroencephalogram (EEG) Cap**. It is equipped with 32 electrodes to capture cerebral electrical activity, and connected to EEG acquisition equipment to ensure data synchronization. **3)** **The** **VR Controller**. Participants use the VR controller to interact with the virtual environment, facilitating manipulation of virtual objects and navigation within the simulation. **4)** **The Display in VR**. A large screen that mirrors the visual content seen by the participant through the VR headset, allowing observers to view the same virtual scenario. **5) EEG Acquisition Equipment**. This records EEG signals from the EEG cap, critical for correlating neural responses with the stimuli presented in the VR environment.


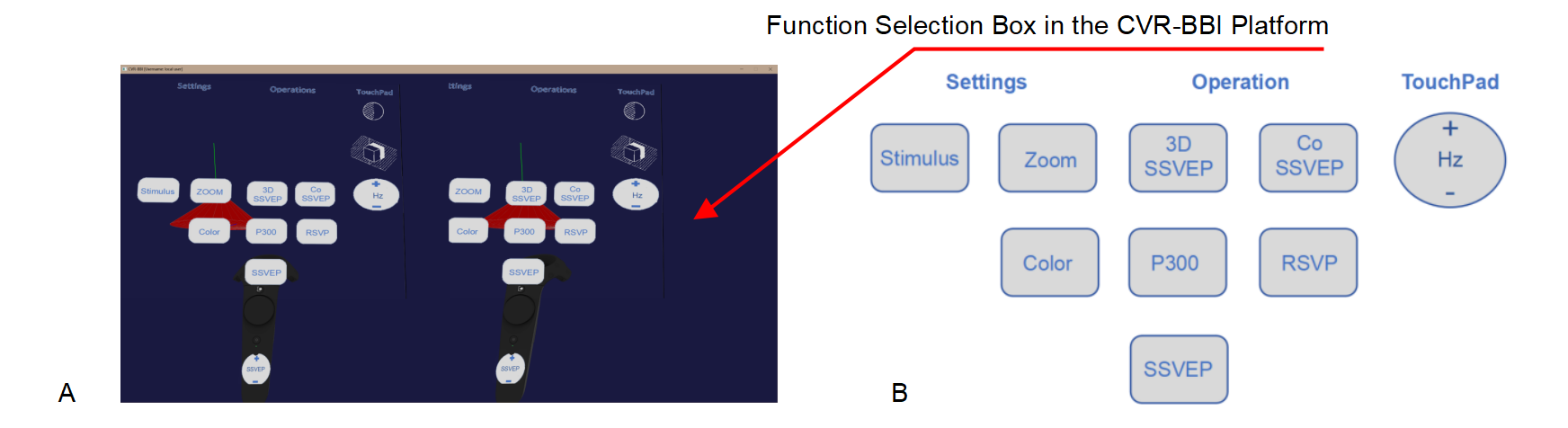


**Fig. S2. The Interface of the CVR-BBI Client.** **A) The VR Interface.** The VR environment presents a dual-view interface, integrating interactive control buttons designed for easy adjustment of stimuli and experimental settings by users. **B) The Function Selection Box**. This panel provides an overview of the layout and options within the CVR-BBI platform. It is equipped with sections for 'Settings', 'Operation', and a 'TouchPad', enabling users to choose EEG paradigms and adjust the parameters of visual stimuli. This interface is designed to ensure intuitive real-time manipulation and customization of experimental parameters.


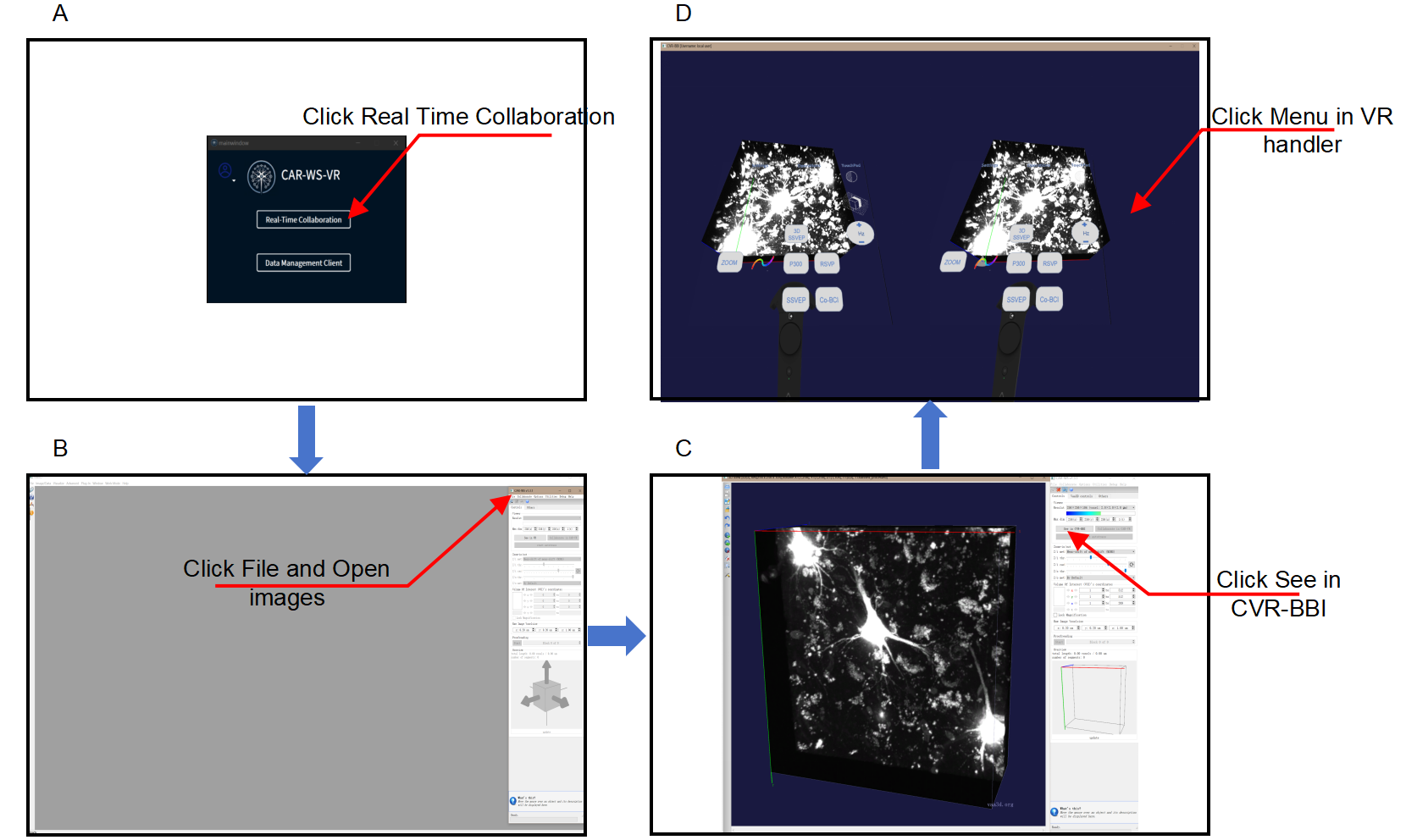


**Fig. S3. CVR-BBI Client Usage Workflow.** **A) Login with Personal Information.** Users initiate the client by logging into the system with their personal credentials, ensuring that all user data and settings are personalized and secure. **B) Select Stimulus Images.** After logging in, users navigate to the image selection interface shown in the top right corner of the figure, where they can choose specific visual stimuli for the experiment by clicking on "File". **C) Enter CVR Mode.** Following image selection, users enter the CVR mode by clicking "See in CVR-BBI" in the interface, also located in the top right corner of the interface. **D) Choose the Paradigm and Start the Experiment.** In CVR mode, users operate the VR controller to access the settings, where they can select an experimental paradigm, and tailor the stimulus parameters to their requirements. Once they have configured their preferences, users initiate the experiment by clicking the start button and continue until its completion.


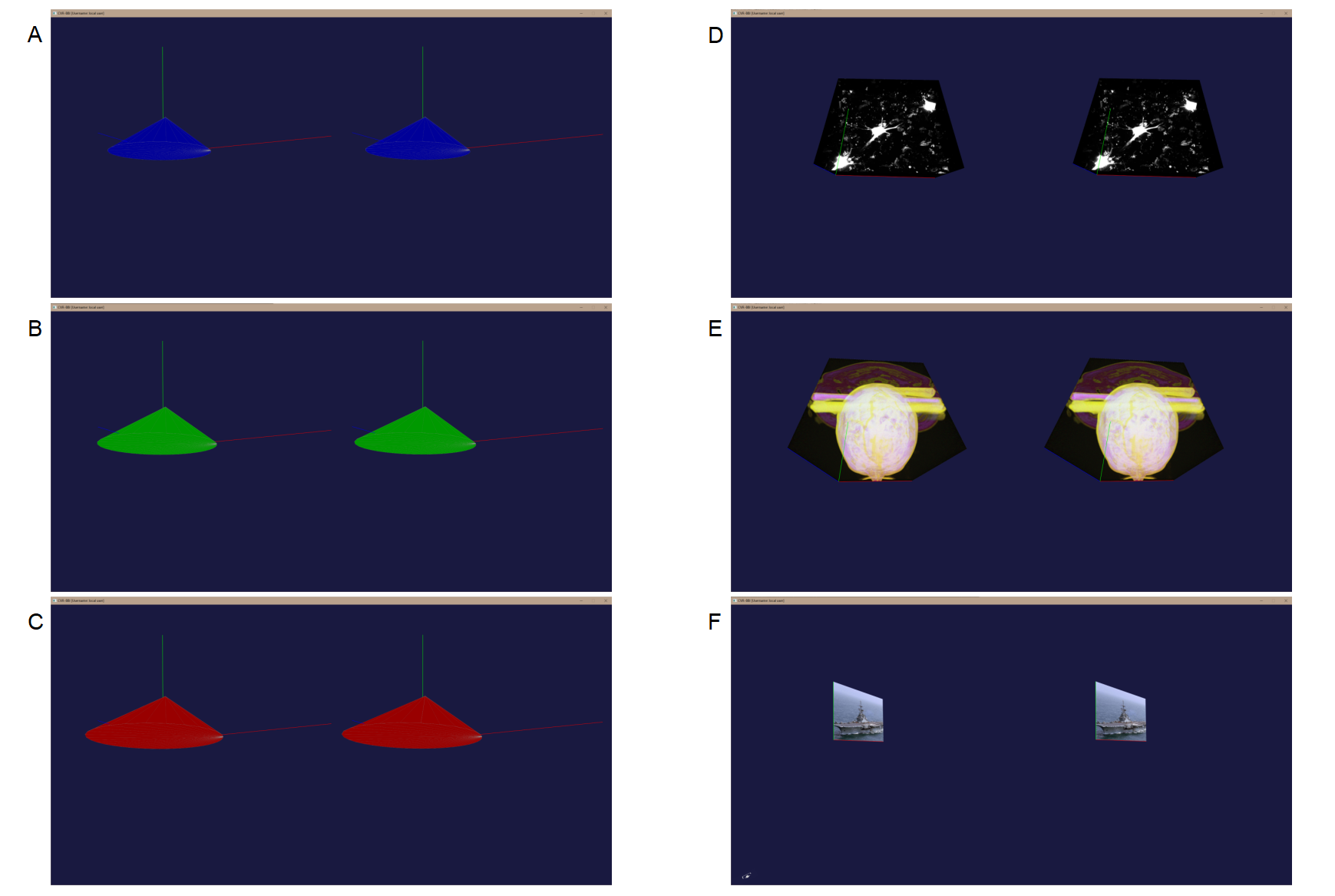


**Fig. S4. Types of Stimuli** **Displayed in VR. A-C) Basic Geometric Shapes.** These panels present three-dimensional rendering of simple geometric shapes, such as cones, designed to evaluate basic visual processing and spatial perception skills. The size, color, and type of geometric shapes can be modified to suit various experimental designs. **D-E) Biomedical Imaging.** These panels present 3D visualizations of neurons and brain imaging, to examine brain responses in tasks such as neuron labeling and other scientific research applications. This type of stimulus plays a vital role in intricate neuroscientific studies. **F) Realistic Natural Scene.** This panel shows an image of an aircraft carrier, demonstrating the platform's capability to integrate complex and specific real-world scenes. This type of stimulus is used in studies on exploring behavioral responses, memory recall tests, or sensory decoding, providing a realistic context for experimental subjects.


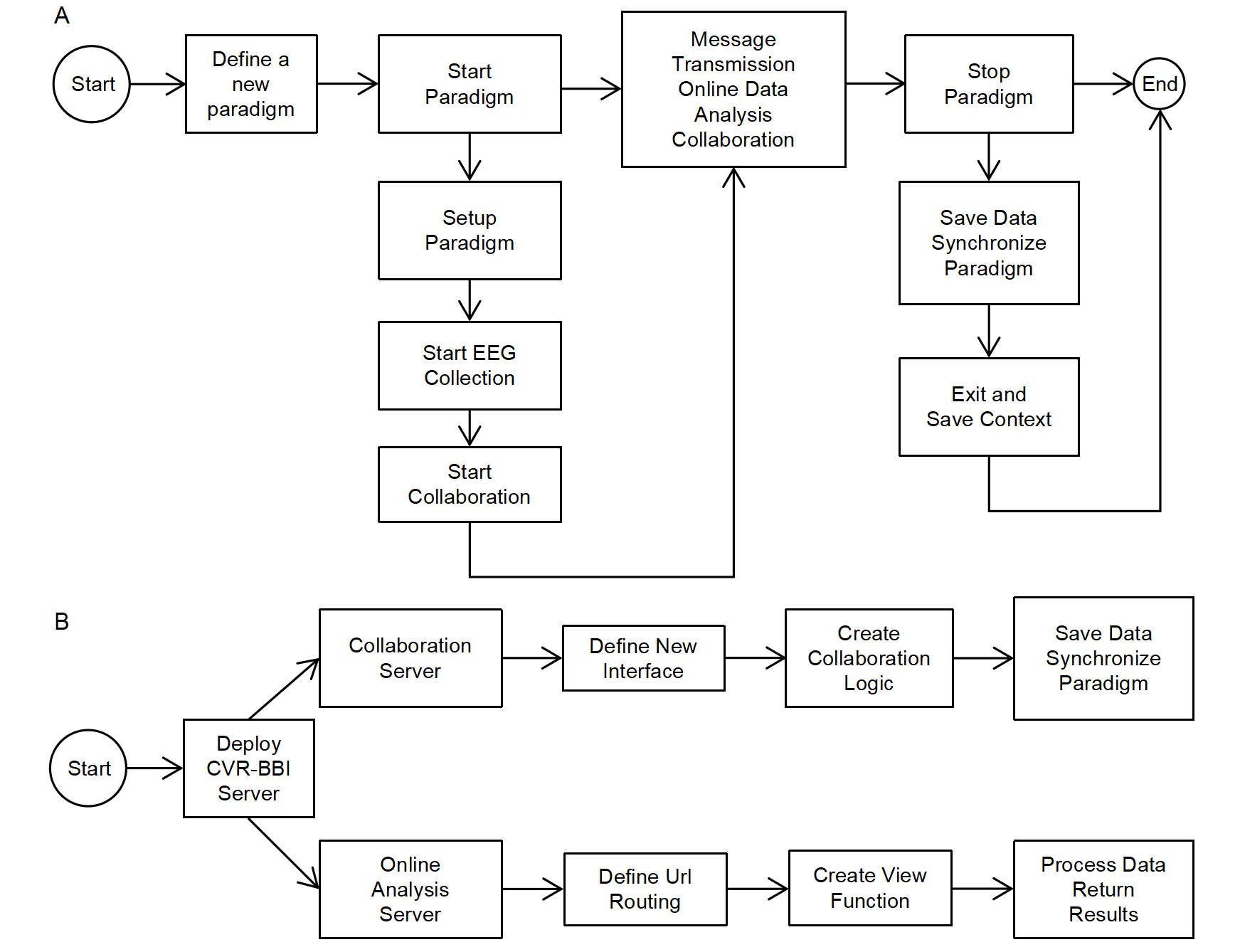


**Fig. S5. The Development Workflow for Customized Paradigms. A) The Operation Flow of the Client. B) The Operation Flow of the Server.**
